# Differential Assessment of the Global Tobacco Index and Smoking Intensity and Duration on Blood Pressure in Peruvian Adults

**DOI:** 10.3390/jcm15031030

**Published:** 2026-01-28

**Authors:** Víctor Juan Vera-Ponce, Fiorella E. Zuzunaga-Montoya, Félix García-Ahumada, Darwin A. León-Figueroa, Percy Díaz Morón, Mario J. Valladares-Garrido

**Affiliations:** 1 Instituto de Investigación de Enfermedades Tropicales, Universidad Nacional Toribio Rodríguez de Mendoza de Amazonas (UNTRM), Amazonas 01001, Peru; vicvepo@gmail.com; 2 Facultad de Medicina (FAMED), Universidad Nacional Toribio Rodríguez de Mendoza de Amazonas (UNTRM), Amazonas 01001, Peru; 3 Universidad Continental, Lima 15049, Peru; fiorellazuzunaga@gmail.com; 4 Escuela de Medicina Humana, Universidad Señor de Sipán, Chiclayo 14001, Peru; felixgarcia@uss.edu.pe (F.G.-A.); percydiaz@uss.edu.pe (P.D.M.); 5 Facultad de Medicina Humana, Universidad de San Martín de Porres, Chiclayo 15011, Peru; darwin_leon@usmp.pe

**Keywords:** tobacco smoking, arterial pressure, smoking reduction, public health, Peru

## Abstract

**Introduction:** Smoking is a known risk factor for cardiovascular diseases, but its specific relationship with blood pressure remains a topic of debate. Recent studies suggest that different aspects of smoking behavior, such as intensity and duration, may have distinct effects on blood pressure. **Objective:** To evaluate the association between different measures of smoking behavior and blood pressure in the Peruvian population. **Methodology:** A cross-sectional analysis was conducted using data from the Peruvian Demographic and Family Health Survey (DHS) from 2018 to 2023. Multiple aspects of smoking were assessed, including current smoking status, intensity, duration, and the global tobacco index. Multiple linear regression models were used to examine the association between these measures and systolic (SBP) and diastolic (DBP) blood pressure, adjusting for sociodemographic and health variables. **Results:** A significant association was found between smoking intensity and elevated blood pressure. Smokers of 20 or more cigarettes per day showed an increase of 6.82 mmHg in SBP (95% CI: 2.90, 10.75) and 5.07 mmHg in DBP (95% CI: 2.44, 7.70) compared to non-smokers. The global tobacco index and smoking duration showed weaker and more variable associations with blood pressure. **Conclusions:** Smoking intensity, rather than other measures of smoking behavior, is more strongly associated with blood pressure levels. These findings highlight the importance of considering smoking intensity in cardiovascular risk assessment and suggest that prevention strategies should focus not only on cessation but also on reducing smoking intensity.

## 1. Introduction

Blood pressure, measured through its systolic (SBP) and diastolic (DBP) components, is a critical indicator of cardiovascular health. Even slight elevations in these values are associated with a significant increase in the risk of cardiovascular and cerebrovascular diseases. In fact, for every 20 mmHg increase in SBP or ten mmHg in DBP, the risk of mortality from coronary heart disease doubles in middle-aged adults [1].

Smoking, a well-established cardiovascular risk factor, affects more than 1.1 billion people worldwide [2]. Its impact on blood pressure has been the subject of numerous studies, often conflicting results. While some researchers have reported acute increases in SBP and DBP immediately after smoking [3], others have observed lower blood pressure values in chronic smokers compared to non-smokers [4].

The complexity of the relationship between smoking and blood pressure is reflected in the considerable variability within the scientific literature on how “smoking status” is defined and operationalized. While most studies have used simple categories such as “current smoker” versus “non-smoker,” recent research suggests that more detailed aspects of smoking behavior are crucial. For example, Linneberg et al. found that heavy cigarette consumption (≥20 per day) was associated with a significant increase in SBP, an effect not observed in lighter smokers [5]. Similarly, a longitudinal study by Doonan et al. demonstrated that the effects of smoking on arterial stiffness, a factor influencing blood pressure, are cumulative and dose-dependent [6].

This heterogeneity in measurement can lead to significant discrepancies in estimating the risk associated with smoking, especially in studies that use smoking status as an adjustment factor. Therefore, examining multiple ways of assessing smoking status is essential, ranging from simple measures of recent consumption to more complex metrics that consider intensity, duration, and cumulative index (pack-years). However, only some studies have thoroughly explored how these different measures of smoking behavior compare in their association with SBP and DBP. A comprehensive approach incorporating these diverse metrics would enable a direct comparison of how different definitions of smoking affect cardiovascular risk assessment, providing a more complete and accurate understanding of this complex relationship.

In the context of middle-income countries such as Peru, where the prevalence of smoking is around 10% in adults [7] and the prevalence of arterial hypertension is 21.07% [8], understanding the relationship between tobacco consumption and blood pressure is crucial for public health. A detailed exploration of how different aspects of smoking behavior are associated with SBP and DBP could provide valuable insights for primary prevention strategies and cardiovascular risk management, especially in populations where healthcare resources are limited.

## 2. Methods

### 2.1. Study Design and Context

This study adopts an observational, cross-sectional, and analytical approach, utilizing secondary data from Peru’s Demographic and Family Health Survey (DHS). The analysis covers six years, from 2018 to 2023, providing a broad and up-to-date perspective on health trends in the Peruvian population [9]. DHS is a nationally recognized survey known for its methodological rigor and capacity to provide critical information on various aspects of public health in Peru.

### 2.2. Population, Sample, and Eligibility Criteria

The target population of this study comprises all adults aged 18 years or older residing in private households in Peru during the 2018–2023 period. The sampling strategy of DHS is characterized by a two-stage, balanced probabilistic, stratified, and independent design. This methodology ensures adequate representativeness at the departmental level and considers the division between urban and rural areas, allowing for a comprehensive analysis of the country’s geographic and socioeconomic diversity.

For our specific analysis of the relationship between smoking habits and blood pressure, all adult participants with complete data on weight, height, waist circumference, and blood pressure measurements were included.

Specific exclusion criteria were applied to ensure data validity and avoid potential biases. Pregnant women were excluded from the analysis due to physiological changes in blood pressure associated with pregnancy. Additionally, cases with extreme or implausible values were removed to ensure the reliability of anthropometric and blood pressure data. Expressly, participants with a body mass index (BMI) less than 10 kg/m^2^ or greater than 70 kg/m^2^, waist circumference less than 40 cm or greater than 150 cm, systolic blood pressure equal to or greater than 250 mmHg, and diastolic blood pressure below 50 mmHg were excluded [10].

### 2.3. Variables and Measurement

The primary dependent variables in this study are Systolic Blood Pressure (SBP) and Diastolic Blood Pressure (DBP).

Regarding the independent variable, a multidimensional approach was employed to assess smoking habits, utilizing nine categories that capture various aspects of tobacco use. These categories range from simple measures of recent consumption to more complex assessments of smoking intensity and duration. Recent consumption was evaluated through questions about smoking within the past 12 months and 30 days. Smoking status was categorized in three different ways, based on whether the participants were “Never smokers,” “Former smokers,” “Current smokers,” or “Daily smokers,” allowing for different levels of classification. Smoking intensity was assessed by distinguishing between smokers of fewer than 20 cigarettes per day and those smoking 20 or more. The Global Tobacco Index (TGI) was calculated by considering both the intensity and duration of smoking, categorizing it into three levels. Finally, the duration of smoking was stratified into six categories, ranging from less than 10 years to 50 years or more. This comprehensive approach enables a detailed analysis of how different aspects of smoking habits are related to blood pressure, capturing not only the current smoking status but also long-term consumption patterns and habit intensity. Moreover, this multiple categorization facilitates comparison with previous studies that have used different definitions of smoking status, thereby contributing to a more complete and nuanced understanding of the relationship between smoking and cardiovascular health.

Demographic variables included sex, categorized as male or female, and age, measured in completed years. Additionally, the area of residence was classified as urban or rural, and the wealth index was stratified into five levels: very poor, poor, medium, rich, and very rich. Alcohol consumption was categorized as “no consumption,” “non-excessive consumption,” and “excessive consumption.” The altitude of the residence, expressed in meters above sea level (m.a.s.l.), was also included. Finally, anthropometric measures such as waist circumference (WC), measured in centimeters, and BMI, calculated as weight in kilograms divided by height in meters squared, were included as measures of overall nutritional status.

### 2.4. Procedures

The DHS in Peru has evolved in terms of data collection methods, transitioning from paper questionnaires to digital tablets in 2016. Information is collected following standardized and internationally validated protocols, with trained personnel from the National Institute of Statistics and Informatics (INEI, acronym in Spanish) conducting interviews and measurements in selected households.

Regularly calibrated automatic digital sphygmomanometers are used for blood pressure measurement. Three blood pressure measurements are taken with the participant seated after a resting period of at least 5 min. The average of the last two measurements is analyzed, discarding the first to minimize the “white coat effect.”

Smoking-related variables were obtained through structured questionnaires. Smoking status, consumption intensity (cigarettes per day), and duration of the habit were recorded by self-report. The tobacco index (pack-years) was subsequently calculated using information on smoking intensity and duration.

Anthropometric measurements are performed with equipment calibrated daily. Weight is measured with SECA model 874 electronic scales (precision 0.1 kg), and height is measured with portable SECA model 213 stadiometers (precision 1 mm), manufactured in Hamburg, Germany. Participants are weighed in light clothing and without shoes, following the Frankfurt protocol for head positioning during height measurement. Waist circumference is measured with a non-stretchable measuring tape (precision 0.1 cm) at the midpoint between the lower edge of the last palpable rib and the top of the iliac crest, with the participant standing and after a normal exhalation.

All anthropometric measurements are taken twice, and the average is used. A third measurement is performed in significant discrepancies (>0.5 kg in weight, >1 cm in height or circumference), and the average of the two closest measurements is used. Finally, information on sociodemographic, economic, and lifestyle variables (including alcohol consumption) is collected through structured questionnaires during the interview.

All participants provide informed consent before participating. The collected data undergo a rigorous quality control process, including direct supervision of a sample of measurements and repetition of measurements in a subsample of participants to ensure the accuracy and reliability of the data.

### 2.5. Statistical Analysis

The statistical analysis was performed using R version 4.1.0, incorporating the ‘survey’ package to handle the complex sampling design of the DHS 2018–2023. The survey design was specified using primary sampling units (PSU, variable QHCLUSTER, which represents the cluster identifier) as clusters, sampling strata (variable HV022), and individual sampling weights. Variance estimation was conducted using Taylor series linearization, the default method in the survey package, which accounts for the complex sampling design. All analyses were weighted using the sampling weights provided by DHS to ensure the national representativeness of the results. For strata with single PSUs, the variance was adjusted using the ‘adjust’ option.

First, a detailed descriptive analysis of all study variables was conducted. Frequencies and percentages were calculated for categorical variables, while for continuous variables, means and standard deviations were obtained. Subsequently, a bivariate analysis was performed to examine the relationship between SBP and DBP with the independent variables and covariates.

Next, multiple linear regression analyses were conducted for each outcome (SBP and DBP) and each type of smoking status assessment. Each smoking metric was examined in a separate model to serve as the primary predictor, with all models adjusted for the same set of covariates (sex, age, area of residence, wealth index, alcohol consumption, BMI, waist circumference, and altitude). For numerical covariates (age, BMI, waist circumference, and altitude), restricted cubic splines (RCS) with three knots were used to capture possible non-linear relationships. The placement of knots was determined automatically by the ‘ns’ function, which positions them at default locations based on the distribution of each variable.

The regression models were fitted using the ‘style’ function from the ‘survey’ package to account for the complex survey design. Regression coefficients, standard errors, and 95% confidence intervals were obtained for each variable in the models. It is important to note that all statistical analyses were performed considering the complex survey design, using functions from the ‘survey’ package such as ‘svymean’, ‘svyquantile’, and ‘svyby’ to obtain weighted estimates and appropriate standard errors. The results were presented in tables and graphs using the ‘gtsummary’ and ‘ggplot2′ packages to provide a clear and detailed visualization of the findings.

### 2.6. Ethical Considerations

This study is based on the secondary analysis of data from DHS, which is publicly accessible and available. The INEI of Peru provides these data without including personal identifiers, thus ensuring participant anonymity. Since there was no direct interaction with the survey subjects and only anonymized data were used, no additional review by an ethics committee was required to conduct this secondary analysis.

However, it is essential to emphasize that this research strictly adhered to the ethical principles established in the Declaration of Helsinki. The research team is committed to preserving data integrity, using it solely for the purposes specified in this study. Furthermore, it has been ensured that the presentation of results maintains the confidentiality and anonymity of the participants.

## 3. Results

Data from 171,129 participants were analyzed in this study, with a balanced distribution by sex (51.64% male). The mean age was 43.20 years (SD: 17.16). The majority of participants resided in urban areas (84.64%) and had secondary (43.63%) or higher education (35.80%). The distribution of the wealth index was uniform across quintiles. Regarding habits, 67.09% reported non-excessive alcohol consumption, while 3.11% reported excessive consumption. The mean BMI was 27.59 kg/m^2^ (SD: 4.76), suggesting a tendency toward overweight in the population. In terms of smoking, 18.51% smoked in the past 12 months, but only 10.58% smoked in the past 30 days. The prevalence of current smokers was 8.99%, with 1.59% being daily smokers. The average blood pressure was 122.57/74.63 mmHg (See Table 1).

**Figure 1:**
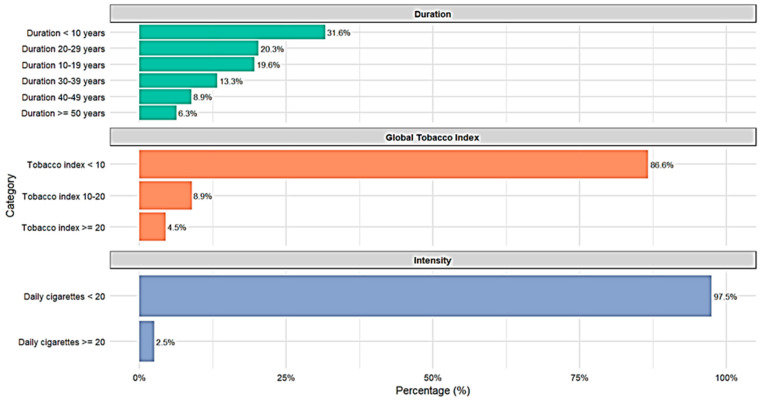
Distribution of Daily Smoker Subtypes.

Analysis of the daily smoker subtypes revealed interesting patterns in terms of intensity, global tobacco index, and duration of the habit. Regarding intensity, most (97.5%) consume fewer than 20 cigarettes daily. The Global Tobacco Index (GTI) shows that 86.6% of daily smokers have an index of less than 10 pack-years, 8.9% between 10 and 20 pack-years, and only 4.5% exceed 20 pack-years. Regarding duration, a more varied distribution is observed: 31.6% smoked for less than 10 years, 19.6% for 10 to 19 years, and 20.3% for 20 to 29 years, with decreasing percentages for longer durations, reaching 6.3% for those who have smoked for 50 years or more (See Figure 1).

Table 2 presents the bivariate, crude, and adjusted distribution between SBP and smoking status. In the adjusted analysis, daily smokers showed an increase of 1.63 mmHg (95% CI: 1.00, 2.26) compared to non-daily smokers. Former and current smokers showed increases of 0.32 mmHg (95% CI: 0.05, 0.58) and 0.51 mmHg (95% CI: 0.26, 0.77), respectively, compared to those who had never smoked. Smoking intensity revealed a marked effect, with smokers of 20 or more cigarettes per day showing an increase of 6.82 mmHg (95% CI: 2.90, 10.75). Regarding duration, a significant effect was observed only in smokers of less than 10 years, with an increase of 1.40 mmHg (95% CI: 0.37, 2.44). Interestingly, smoking within the last 12 months has been associated with a decrease of 0.29 mmHg (95% CI: −0.49, −0.09).

Table 3 presents the bivariate, crude, and adjusted distribution between DBP and smoking status. In the adjusted analysis, daily smokers showed an increase of 0.80 mmHg (95% CI: 0.39, 1.20) in DBP compared to non-daily smokers. Smoking intensity revealed a marked effect, with smokers of 20 or more cigarettes per day showing an increase of 5.07 mmHg (95% CI: 2.44, 7.70). The global tobacco index also showed significant associations, with increases of 2.43 mmHg (95% CI: 0.91, 3.95) for an index of 10–20 and 2.53 mmHg (95% CI: 0.26, 4.80) for an index of ≥20. Regarding duration, a significant effect was observed in smokers of 30–39 years, with an increase of 2.63 mmHg (95% CI: 1.43, 3.83). Interestingly, no significant associations were found for former or current smokers in general, suggesting that the impact on DBP may be more related to the intensity and duration of the habit than to the current smoking status.

## 4. Discussion

### 4.1. Main Findings

This study provides an intriguing perspective on the complex relationship between smoking habits and blood pressure in the Peruvian population. By examining multiple aspects of smoking, including intensity, duration, and cumulative index, we found association patterns that go beyond the simple smoker/non-smoker dichotomy. The results highlight the importance of considering smoking as a multidimensional risk factor in cardiovascular health, where both the intensity and chronicity of the habit are strongly associated with blood pressure variations. Indeed, the variability observed in the effects on SBP and DBP according to different smoking metrics suggests distinct underlying mechanisms. These findings challenge the notion of a uniform effect of smoking on blood pressure and underscore the need for a more nuanced approach to cardiovascular risk assessment. Specifically, identifying specific thresholds of intensity and duration associated with significant increases in blood pressure could have important implications for prevention strategies and clinical management.

A distinctive aspect of this study is the comprehensive inclusion of various ways to assess smoking status, ranging from simple measures such as consumption in the last 12 or 30 days to more complex metrics like daily intensity, the global tobacco index, and the duration of the habit. This multifaceted approach addresses the variability observed in the scientific literature regarding how “smoking status” is defined and operationalized, especially in studies that use this variable as an adjustment factor. The findings demonstrate that the choice of smoking measure is not trivial; on the contrary, it significantly influences risk estimation. For example, while the simple categorization of current smokers showed modest effects, considering consumption intensity revealed substantially higher risks for heavy smokers. This distinction is crucial, as it highlights that indiscriminate use of a single definition of smoking status may lead to underestimating or overestimating its impact on cardiovascular health. Moreover, it emphasizes the importance of differentiating between measures appropriate for public health, which may benefit from broader categorizations, and those necessary for precise individual risk assessment, where the intensity and duration of the habit play a fundamental role. This nuanced approach provides a more comprehensive and accurate understanding of the relationship between smoking and blood pressure, offering implications for both epidemiological research and clinical practice.

From a clinical perspective, it is important to distinguish between statistically significant associations and those with meaningful clinical impact. While several smoking categories showed statistically significant associations with blood pressure, the magnitude of these effects varied considerably. For instance, former smokers and current light smokers showed increases of less than 1 mmHg in SBP, which, although statistically significant given our large sample size, are unlikely to translate into substantial cardiovascular risk at the individual level. In contrast, heavy smokers (≥20 cigarettes/day) demonstrated increases exceeding 5 mmHg in both SBP and DBP. Given that each 10 mmHg increase in SBP is associated with a doubling of cardiovascular mortality risk, the 6.82 mmHg elevation observed in heavy smokers represents a clinically meaningful increase that warrants serious consideration in risk stratification and prevention strategies.

### 4.2. Comparison with Previous Studies

Accurate evaluation of smoking status concerning blood pressure is crucial for understanding the cardiovascular risks associated with smoking. In our study, we chose to use blood pressure as a continuous numerical variable rather than applying categorical cutoffs. This decision was based on the constant evolution of clinical guidelines, as evidenced by changes between the 2017 and 2020 guidelines for hypertension [11,12]. Using a continuous scale allows for capturing subtle variations in blood pressure that might be missed with fixed categories, providing a more nuanced picture of the relationship between smoking and blood pressure. Although guidelines often cite increments of 20 mmHg in systolic blood pressure as clinically relevant thresholds for increased cardiovascular risk, treating blood pressure as a continuous variable may be more useful for detecting associations, as cardiovascular risk increases progressively across the entire blood pressure spectrum without abrupt transitions [1].

Our findings reveal that the risk of elevated blood pressure increases specifically with the intensity of the smoking habit. This result is particularly notable given the multiple ways to evaluate smoking status. While more sophisticated measures, such as the GTI, have proven helpful in predicting specific health outcomes, our study suggests that daily consumption intensity is the most determining factor for blood pressure. For example, a study found that the global smoking index was a robust predictor for multiple non-communicable diseases, but its association with blood pressure was not as pronounced [13].

Recent literature has revealed discrepancies in how different smoking statuses are associated with elevated blood pressure. While some studies have found positive associations between any form of smoking and elevated blood pressure [14], others have observed more pronounced effects only in heavy or long-term smokers [15]. These inconsistencies underscore the need for a more detailed approach to evaluating smoking status, considering not only the presence or absence of the habit but also its intensity, duration, and consumption patterns.

The Framingham Heart Study, a cornerstone in cardiovascular research, has provided valuable insights into different ways of evaluating smoking status and its impact on cardiovascular health. Mannan et al. analyzed data from this study and found that both smoking intensity and duration were significant predictors of cardiovascular disease but with differential effects [16]. This finding aligns with our observation that smoking intensity is a stronger predictor of elevated blood pressure than other measures of smoking habits.

However, not all studies have reached the same conclusions. Hackshaw et al., in a meta-analysis of 141 cohort studies, found that even smoking one cigarette a day carried a significant cardiovascular risk [17]. On the other hand, the Vienna Hairdresser Initiative, a pilot screening study in a non-medical setting, identified hypertension through automated office blood pressure measurements in 193 participants. This study reported that 65.8% of participants were hypertensive, with 74.8% receiving no treatment and 63% being unaware of their elevated blood pressure [13]. This approach, similar in some respects to our use of the global tobacco index, found a strong association between smoking and myocardial infarction risk. However, our study suggests that, specifically for blood pressure, current smoking intensity may be more relevant than cumulative exposure.

### 4.3. Public Health Significance

Our study highlights the importance of considering smoking intensity in cardiovascular disease prevention strategies. The findings suggest that public health interventions should not only focus on complete smoking cessation but also on reducing smoking intensity. This aligns with the harm reduction concept proposed by Hatsukami et al., which suggests that even a reduction in the number of daily cigarettes can have significant cardiovascular health benefits [18]. Therefore, public health campaigns could incorporate messages about reducing daily consumption as an intermediate step toward complete cessation.

Additionally, our study’s results directly affect improving cardiovascular risk assessment in primary care. By demonstrating the importance of smoking intensity on blood pressure, our findings suggest that cardiovascular risk assessment tools, such as the Framingham Risk Score or SCORE, could benefit from a more detailed categorization of smoking habits.

Moreover, these findings support the need for more nuanced, evidence-based tobacco control policies. While many current policies focus on preventing smoking initiation and promoting complete cessation, our results suggest that it could also be beneficial to implement strategies aimed at reducing smoking intensity among current smokers. This would align with the World Health Organization’s recommendations on using tobacco taxes and other economic measures to discourage heavy consumption [19]. Furthermore, our findings could inform the development of stricter regulations on nicotine content in cigarettes, as proposed by Donny et al. [20].

This study opens new avenues for public health research related to smoking and cardiovascular health. It suggests the need for longitudinal studies examining how changes in smoking intensity over time affect blood pressure and other cardiovascular risk factors. Additionally, our findings highlight the importance of investigating the biological mechanisms underlying the relationship between smoking intensity and blood pressure. This could lead to the development of more targeted and effective interventions to reduce cardiovascular risk in smokers.

Our study also underscores the need to investigate how these findings might apply to new forms of nicotine consumption, such as electronic cigarettes, an area that Benowitz and Fraiman have identified as crucial for future public health [21].

Finally, our study suggests that the notion of “smoking and that’s it” is an oversimplification when predicting specific health risks, whether it is included to assess direct risk or as a confounding factor. Our findings indicate that the relationship between smoking and cardiovascular outcomes is more nuanced, potentially differing from other diseases, such as lung cancer [22].

### 4.4. Strengths and Limitations of the Study

This study has several notable strengths, including its large sample size and the Peruvian population’s national representativeness, allowing for the generalization of the results. The comprehensive evaluation of smoking habits, considering multiple aspects such as intensity, duration, and cumulative index, provides a more complete understanding of the relationship between smoking and blood pressure. Additionally, using models adjusted for multiple relevant covariates strengthens the validity of our findings.

However, the study also has significant limitations. Its cross-sectional design prevents us from establishing definitive causal relationships between smoking and blood pressure. The reliance on self-reported data regarding smoking habits, without biochemical verification (e.g., cotinine, exhaled CO), may introduce recall bias or social desirability bias, a limitation inherent to secondary analysis of large-scale surveys. Furthermore, the need for biochemical measures of tobacco exposure, such as nicotine levels, limits the objective validation of smoking status. Finally, although multiple confounding factors were controlled for, residual confounding from unmeasured or unknown factors cannot be completely ruled out. These limitations underscore the need for longitudinal studies with more objective measures of smoking to confirm and expand our findings.

## 5. Conclusions

This study found that smoking intensity shows a stronger association with blood pressure compared to other measures of smoking behavior in the Peruvian population. Our findings show that heavy smokers, particularly those who consume 20 or more cigarettes per day, have a significantly higher risk of elevated blood pressure compared to non-smokers and less intense smokers. Additionally, the study reveals that different aspects of smoking behavior, such as duration and the global tobacco index, have distinct effects on SBP and DBP. These results underscore the complexity of the relationship between smoking and cardiovascular health and highlight the importance of considering not only smoking status but also specific consumption patterns in cardiovascular risk assessment.

Based on our findings, it is recommended that smoking prevention and control strategies in Peru and similar settings focus not only on complete cessation but also on reducing smoking intensity as an intermediate goal. Cardiovascular risk assessment programs should incorporate more detailed measures of smoking habits, with particular attention to consumption intensity. It is suggested that local clinical guidelines for hypertension management consider heavy smoking as an additional risk factor that requires closer monitoring. Furthermore, longitudinal studies are recommended to examine how changes in smoking intensity over time affect blood pressure and other cardiovascular risk factors.

## Figures and Tables

**Table 1:** Characteristics of the Study Population.

Characteristic	*n* = 171,129
**Sex**	
Female	82,756 (48.36%)
Male	88,373 (51.64%)
**Age**	43.20 (17.16)
**Residence**	
Urban	144,837 (84.64%)
Rural	26,292 (15.36%)
**Education**	
No Level	351 (0.21%)
Primary	33,631 (20.35%)
Secondary	72,093 (43.63%)
Superior	59,155 (35.80%)
**Wealth Index**	
The poorest	31,252 (18.26%)
Poor	34,453 (20.13%)
Medium	35,690 (20.86%)
Rich	35,042 (20.48%)
The richest	34,692 (20.27%)
**Alcohol Consumption**	
Never or did not use in the last 12 months	50,994 (29.80%)
Non-excessive	114,806 (67.09%)
Excessive	5328 (3.11%)
**Body Mass Index**	27.59 (4.76)
**Waist Circumference**	93.34 (11.68)
**Systolic arterial pressure**	122.57 (18.20)
**Diastolic arterial pressure**	74.63 (10.42)
**Have you smoked in the last 12 months?**	
No	139,454 (81.49%)
Yes	31,675 (18.51%)
**Have you smoked in the last 30 days?**	
No	153,015 (89.42%)
Yes	18,114 (10.58%)
**Smoking Status**	
Has never smoked	139,454 (81.49%)
Former smoker	13,561 (7.92%)
Currently smoker	15,390 (8.99%)
Daily smoker	2723 (1.59%)

**Table 2:** Bivariate, Crude, and Adjusted Analysis of the Relationship between SBP and Smoking Status.

Characteristic	Systolic Blood Pressure	Simple		Multiple	
	(Mean ± SD)	cPR	95% CI	aPR	95% CI
**Have you smoked in the last 12 months?**					
No	116.56 (15.57)	Ref.		Ref.	
Yes	121.29 (14.26)	4.73	(4.53, 4.94)	−0.29	(−0.49, −0.09)
**Have you smoked in the last 30 days?**					
No	116.89 (15.49)	Ref.		Ref.	
Yes	121.87 (14.44)	4.99	(4.73, 5.25)	0.06	(−0.19, 0.30)
**Daily Smoker**					
No	117.30 (15.44)	6.21	(5.50, 6.91)	**1.63**	**(1.00, 2.26)**
Yes	123.50 (15.83)				
**Smoking Status I**					
Has never smoked	116.56 (15.57)	Ref.		Ref.	
Former smoker	120.53 (13.98)	3.97	(3.67, 4.26)	**0.32**	**(0.05, 0.58)**
Currently smoker	121.64 (14.21)	5.08	(4.80, 5.36)	**0.51**	**(0.26, 0.77)**
Daily smoker	123.50 (15.83)	6.95	(6.25, 7.65)	0.72	(0.10, 1.33)
**Smoking Status II**					
Has never smoked	116.56 (15.57)	Ref.		Ref.	
Former smoker	120.53 (13.98)	3.97	(3.67, 4.26)	0.32	(0.05, 0.59)
Currently/Daily smoker	121.87 (14.44)	5.32	(5.06, 5.58)	0.54	(0.30, 0.78)
**Smoking Status III**					
Has never smoked	116.56 (15.57)	Ref.		Ref.	
Former/Current/Daily smoker	121.29 (14.26)	4.73	(4.53, 4.94)	**0.44**	**(0.25, 0.64)**
**Intensity of smoking**					
Has never smoked	116.56 (15.57)	Ref.		Ref.	
Former smoker	120.53 (13.98)	3.97	(3.67, 4.26)	**0.32**	**(0.05, 0.58)**
Currently smoker	121.64 (14.21)	5.08	(4.80, 5.36)	**0.51**	**(0.26, 0.77)**
Daily cigarettes < 20	123.28 (15.61)	6.73	(6.02, 7.44)	0.53	(−0.10, 1.15)
Daily cigarettes ≥ 20	129.53 (19.96)	12.98	(8.54, 17.41)	**6.82**	**(2.90, 10.75)**
**Global Tobacco Index**					
Has never smoked	116.56 (15.57)	Ref.		Ref.	
Former smoker	120.53 (13.98)	3.97	(3.67, 4.26)	**0.32**	**(0.05, 0.58)**
Currently smoker	121.64 (14.21)	5.08	(4.80, 5.36)	**0.51**	**(0.26, 0.77)**
Tobacco index < 10	123.14 (15.27)	6.59	(5.83, 7.34)	0.6	(−0.05, 1.26)
Tobacco index ≥ 10 to <20	127.52 (18.34)	10.96	(8.31, 13.61)	0.88	(−1.39, 3.15)
Tobacco index ≥ 20	130.87 (20.21)	14.32	(10.36, 18.27)	2.52	(−0.86, 5.90)
**Duration of smoking**					
Has never smoked	116.56 (15.57)	Ref.		Ref.	
Former smoker	120.53 (13.98)	3.97	(3.67, 4.26)	**0.32**	**(0.05, 0.59)**
Currently smoker	121.64 (14.21)	5.08	(4.80, 5.36)	**0.52**	**(0.26, 0.77)**
<10 years	121.54 (14.23)	4.98	(3.77, 6.19)	**1.40**	**(0.37, 2.44)**
10–19 years	122.49 (14.29)	5.93	(4.52, 7.34)	1.17	(−0.04, 2.38)
20–29 years	123.18 (15.82)	6.62	(4.92, 8.33)	−0.15	(−1.61, 1.31)
30–39 years	127.62 (18.10)	11.06	(8.97, 13.16)	1.09	(−0.70, 2.89)
40–49 years	128.85 (19.08)	12.29	(9.67, 14.91)	−1.51	(−3.76, 0.73)
≥50 years	132.89 (18.12)	16.33	(12.91, 19.76)	−1.01	(−3.95, 1.93)

Each smoking status variable has been independently adjusted for sex, age (rcs), area of residence, wealth index, alcohol consumption, body mass index (rcs), waist circumference (rcs), and altitude (rcs). cRP: Crude Prevalence ratio. aRP: Adjusted Prevalence ratio. 95% CI: 95% confidence interval. Source: self-made.

**Table 3:** Bivariate, Crude, and Adjusted Analysis of the Relationship between DBP and Smoking Status.

Characteristic	Diastolic Blood Pressure	Simple		Multiple	
	(Mean ± SD)	cPR	95% CI	aPR	95% CI
**Have you smoked in the last 12 months?**					
No	72.57 (9.52)	Ref.		Ref.	
Yes	74.68 (9.76)	2.11	(1.98, 2.24)	−0.09	(−0.22, 0.04)
**Have you smoked in the last 30 days?**					
No	72.72 (9.54)	Ref.		Ref.	
Yes	74.93 (9.82)	2.21	(2.05, 2.37)	0.09	(−0.07, 0.24)
**Daily Smoker**					
No	72.91 (9.58)	Ref.		Ref.	
Yes	75.43 (10.57)	2.53	(2.09, 2.96)	0.8	(0.39, 1.20)
**Smoking Status II**					
Has never smoked	72.57 (9.52)	Ref.		Ref.	
Former smoker	74.36 (9.66)	1.79	(1.61, 1.97)	−0.05	(−0.23, 0.13)
Currently smoker	74.86 (9.71)	2.28	(2.11, 2.46)	0.08	(−0.09, 0.26)
Daily smoker	75.43 (10.57)	2.86	(2.42, 3.30)	0.53	(0.12, 0.94)
**Smoking Status II**					
Has never smoked	72.57 (9.52)	Ref.		Ref.	
Former smoker	74.36 (9.66)	1.79	(1.61, 1.97)	−0.05	(−0.23, 0.13)
Currently/Daily smoker	74.93 (9.82)	2.36	(2.19, 2.52)	0.14	(−0.02, 0.30)
**Smoking Status III**					
Has never smoked	72.57 (9.52)	Ref.		Ref.	
Former/Current/Daily smoker	74.68 (9.76)	2.11	(1.98, 2.24)	0.06	(−0.08, 0.19)
**Intensity of smoking**					
Has never smoked	72.57 (9.52)	Ref.		Ref.	
Former smoker	74.36 (9.66)	1.79	(1.61, 1.97)	−0.05	(−0.23, 0.13)
Currently smoker	74.86 (9.71)	2.28	(2.11, 2.46)	0.08	(−0.09, 0.26)
Daily cigarettes < 20	75.30 (10.36)	2.73	(2.28, 3.17)	0.4	(−0.02, 0.82)
Daily cigarettes ≥ 20	79.66 (16.05)	7.09	(4.33, 9.85)	5.07	(2.44, 7.70)
**Global Tobacco Index**					
Has never smoked	72.57 (9.52)	Ref.		Ref.	
Former smoker	74.36 (9.66)	1.79	(1.61, 1.97)	−0.05	(−0.23, 0.13)
Currently smoker	74.86 (9.71)	2.28	(2.11, 2.46)	0.08	(−0.09, 0.26)
Tobacco index < 10	75.26 (10.34)	2.69	(2.22, 3.16)	0.26	(−0.17, 0.70)
Tobacco index ≥ 10 to <20	77.38 (11.86)	4.8	(3.15, 6.45)	2.43	(0.91, 3.95)
Tobacco index ≥ 20	77.42 (13.72)	4.85	(2.39, 7.31)	2.53	(0.26, 4.80)
**Duration of smoking**					
Has never smoked	72.57 (9.52)	Ref.		Ref.	
Former smoker	74.36 (9.66)	1.79	(1.61, 1.97)	−0.05	(−0.23, 0.13)
Currently smoker	74.86 (9.71)	2.28	(2.11, 2.46)	0.08	(−0.09, 0.26)
<10 years	74.16 (10.13)	1.59	(0.83, 2.34)	0.15	(−0.55, 0.84)
10–19 years	75.79 (10.34)	3.22	(2.34, 4.10)	0.41	(−0.40, 1.22)
20–29 years	76.43 (11.12)	3.86	(2.80, 4.92)	0.37	(−0.61, 1.35)
30–39 years	78.46 (10.78)	5.89	(4.59, 7.19)	2.63	(1.43, 3.83)
40–49 years	75.27 (10.98)	2.69	(1.06, 4.32)	0.05	(−1.46, 1.55)
≥50 years	73.46 (10.92)	0.89	(−1.25, 3.02)	−0.34	(−2.31, 1.63)

Each smoking status variable has been independently adjusted for sex, age (rcs), area of residence, wealth index, alcohol consumption, body mass index (rcs), waist circumference (rcs), and altitude (rcs). cRP: Crude Prevalence ratio. aRP: Adjusted Prevalence ratio. 95% CI: 95% confidence interval. Source: self-made.

## Data Availability

The data supporting the findings of this study can be accessed by the original research paper at the follow link: https://proyectos.inei.gob.pe/microdatos/.
